# Impact of a Nosocomial COVID-19 Outbreak on a Non-COVID-19 Nephrology Ward during the First Wave of the Pandemic in Spain

**DOI:** 10.3390/antibiotics10060619

**Published:** 2021-05-22

**Authors:** María Milagro Montero, Carlota Hidalgo López, Inmaculada López Montesinos, Luisa Sorli, Cristina Barrufet Gonzalez, Judith Villar-García, Roberto Güerri-Fernández, Milagros Herranz, Marta Crespo, María Dolores Arenas Jiménez, Julio Pascual, Cristina González Juanes, Juan P. Horcajada

**Affiliations:** 1 Infectious Diseases Service, Hospital del Mar, Institut Hospital del Mar d’Investigacions Mèdiques (IMIM), Universitat Autònoma de Barcelona (UAB), CEXS-Universitat Pompeu Fabra, 08003 Barcelona, Spain; ilopezmontesinos@parcdesalutmar.cat (I.L.M.); lsorli@parcdesalutmar.cat (L.S.); jvillar@parcdesalutmar.cat (J.V.-G.); rguerri@parcdesalutmar.cat (R.G.-F.); 2 Health Services Evaluation and Clinical Epidemiology Department, Institut Hospital del Mar d’Investigacions Mèdiques (IMIM), Hospital del Mar, 08003 Barcelona, Spain; chidalgo@parcdesalutmar.cat (C.H.L.); cbarrufet@parcdesalutmar.cat (C.B.G.); mherranz@parcdesalutmar.cat (M.H.); cgonzalez@parcdesalutmar.cat (C.G.J.); 3 Department of Nephrology, Hospital del Mar, 08003 Barcelona, Spain; mcrespo@parcdesalutmar.cat (M.C.); marenasjimenez@parcdesalutmar.cat (M.D.A.J.); jpascual@parcdesalutmar.cat (J.P.)

**Keywords:** nosocomial outbreak, COVID-19, infection control

## Abstract

Introduction: The aim of this study was to analyze a nosocomial coronavirus disease 2019 (COVID-19) outbreak that occurred on a polyvalent non-COVID-19 ward at a tertiary care university hospital in Spain during the first wave of the pandemic and to describe the containment measures taken. The outbreak affected healthcare workers (HCWs) and kidney disease patients including transplant patients and those requiring maintenance hemodialysis. Methods: The outbreak investigation and report were conducted in accordance with the Orion statement guidelines. Results: In this study, 15 cases of COVID-19 affecting 10 patients and 5 HCWs were identified on a ward with 31 beds and 43 HCWs. The patients had tested negative for severe acute respiratory syndrome coronavirus 2 infection on admission. One of the HCWs was identified as the probable index case. Five patients died (mortality rate, 50%). They were all elderly and had significant comorbidities. The infection control measures taken included the transfer of infected patients to COVID-19 isolation wards, implementation of universal preventive measures, weekly PCR testing of patients and HCWs linked to the ward, training of HCWs on infection control and prevention measures, and enhancement of cleaning and disinfection. The outbreak was contained in 2 weeks, and no new cases occurred. Conclusion: Nosocomial COVID-19 outbreaks can have high attack rates involving both patients and HCWs and carry a high risk of patient mortality. Hospitals need to implement effective infection prevention and control strategies to prevent nosocomial COVID-19 spread.

## 1. Introduction

Coronavirus disease 19 (COVID-19), caused by severe acute respiratory syndrome coronavirus 2 (SARS-CoV-2), was first identified in Wuhan, China, in December 2019 and subsequently spread to 188 countries around the world [1]. Since 31 December 2019 and as of April 2021, 147,443,848 cases of COVID-19 and 3,117,542 deaths have been reported. A substantial number of the cases—49,599,293—correspond to Europe [2].

According to recent data from the Catalan Health Department, there were 633,227 COVID-19 cases and 21,895 deaths in Catalonia as of 5 May 2021 [3]. The cumulative number of cases at our hospital was 3194 and 900 of these had occurred during the first week of April 2020.

COVID-19 is a threat in hospital settings since it can cause large outbreaks among healthcare workers (HCWs) [4] and patients, clearly posing unique infection prevention and control (IPC) challenges [5].

Several outbreaks were initially reported in Wuhan, including one at a university hospital with 138 cases, 41% of which were suspected to be nosocomial (17 patients and 40 HCWs) [6,7,8]. According to a recent report, one in five confirmed cases of COVID-19 in the United States involves an HCW [9].

There is sufficient evidence to support the hypothesis that SARS-CoV-2 is transmitted primarily by droplets that remain airborne following aerosol-generating procedures [10,11,12]. The disease can also be transmitted by asymptomatic or presymptomatic individuals [13,14,15]. Since hospitals are often epicenters of newly circulating infections, HCWs are at risk, both at work and in the community [7,16].

Enhancement of IPC measures, such as the creation of separate COVID-19 and non-COVID-19 areas, is essential for reducing hospital outbreaks [17,18]. During the first wave of the pandemic, a large part of our hospital was converted into a COVID-19 facility. This process involved major structural and organizational changes and created difficulties in the management of COVID-19-free areas.

Patients with kidney disease, both those requiring maintenance hemodialysis and transplant recipients, are at high risk for COVID-19 and a poor outcome. Preventive measures, thus, are crucial [19,20].

In this article, we report on a nosocomial COVID-19 outbreak that affected HCWs and patients on a nephrology ward at a tertiary care university hospital in Spain and describe the infection control measures implemented. The outbreak was associated with a high attack rate and high patient mortality.

## 2. Materials and Methods

In preparing this report, we followed the ORION statement guidelines for transparent reporting of outbreak reports and intervention studies of nosocomial infection [21].

### 2.1. Institutional Setting and Study Period

This study was conducted at Hospital del Mar in Barcelona, Spain from 6 April to 30 May 2020. Hospital de Mar is a tertiary care hospital with 413 beds and 16 hospitalization units; it has 4545 HCWs, including 929 physicians and residents, 1332 nurses, and 997 care assistants.

All patients admitted to the hospital during the study period were screened per protocol for COVID-19 using reverse transcriptase–polymerase chain reaction (PCR). Depending on the result, they were assigned to a COVID-19 or a non-COVID-19 ward. HCWs were not routinely tested during this period unless they had compatible clinical symptoms. Access to the hospital was exclusively restricted to HCWs. Family visits were forbidden.

The COVID-19 outbreak occurred on a polyvalent hospitalization ward that provides care to general surgery, nephrology, and kidney transplant patients.

### 2.2. Study Objectives

The aim of this study was to describe a nosocomial COVID-19 outbreak in a polyvalent non-COVID-19 hospital unit and to report on the measures taken to detect and control the outbreak.

### 2.3. Variables and Data Sources

Demographic, clinical, laboratory, and epidemiological data were collected from hospital and primary care electronic medical records and included the following: age, sex, presence, and severity of comorbidities assessed using the Charlson comorbidity index [22], days from exposure to positive PCR test for COVID-19, clinical symptoms, chest X-ray findings, treatment of COVID-19, and outcomes. Individuals were followed until death or for at least 30 days from the first positive test.

### 2.4. Clinical Classification of COVID-19 and Definition of Case

Individuals were recorded as cases if at least one of the following tests was interpreted as positive for SARS-CoV-2: PCR or rapid diagnostic test based on antigen detection in a sample taken from the respiratory tract (throat swab, sputum, endotracheal aspirate, or bronchoalveolar lavage fluid). Contacts were identified based on potential exposure to a probable index case within 48 h of symptom onset. Close contact was defined as being within 6 feet of an infected person for 15 consecutive minutes or more.

For the purpose of the investigation, cases were classified as probably hospital-acquired if the patient developed COVID-19 symptoms (or had their first positive PCR test) between day 8 and 14 of admission and as definitely hospital-acquired if they developed symptoms or tested positive later.

On detection of the outbreak, patients and HCWs were interviewed daily over a period of 14 days to check for typical COVID-19-symptoms. Universal PCR testing was also performed among all patients on the affected ward and all HCWs linked to the ward to detect asymptomatic cases.

### 2.5. Intervention Methods

The following measures were taken to evaluate the hospital’s strategy for the early detection and containment of potential COVID-19 outbreaks:

- Review of medical files, laboratory records, and radiographic findings of confirmed cases;
- Review of inpatient electronic tracking system to determine the movement of cases within and between units and to identify the exact location of beds occupied;
- Scrutiny of staff records to understand work areas and shift patterns of infected staff;
- Personal and telephone interviews with HCWs involved in the care of affected patients and preparation of a contact list for PCR studies;
- Closure of affected ward with no admission for the first 2 weeks of the outbreak;
- Banning of transfers to other wards;
- Weekly PCR testing of all patients and HCWs (including cleaning staff) in the affected ward;
- Mandatory confinement for HCWs with a positive PCR or who were close contacts with positive cases as per the hospital protocol;
- Transfer of patients with a positive PCR to COVID-19 isolation wards;
- Daily safety meeting with all staff involved in the outbreak;
- Detailed analyses of human behaviors and interactions, room sizes, and ventilation characteristics of the ward and common areas.

### 2.6. Statistical Analysis

Continuous quantitative variables are presented as the median and interquartile range (IQR). Continuous variables were compared using the *t* test or Mann–Whitney U test and categorical variables using the Fisher’s exact test or Pearson’s χ^2^ test, as appropriate. Analysis of variance was used to determine if the means of groups were significantly different from each other. All *p*-values were two tailed, and statistical significance was set at <0.05. Statistical analyses were performed in SPSS V. 21.0 (SPSS Inc., Chicago, IL, USA).

## 3. Results

### 3.1. Epidemiological Curve

**Figure 1:**
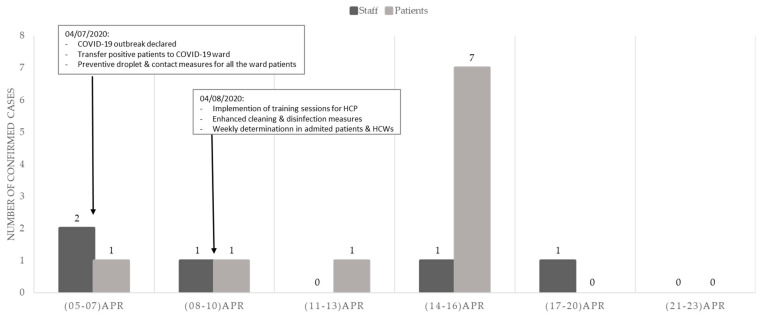
Epidemiological curve: COVID-19 coronavirus disease 2019; HCW healthcare workers; ICP infection control and prevention.

Over an 8-week study period (6 April to 31 May), there were 15 confirmed COVID-19 cases: 10 in patients and 5 in HCWs. The epidemiological curve is shown in Figure 1.

### 3.2. Case Detection and Epidemiological Investigation

**Figure 2:**
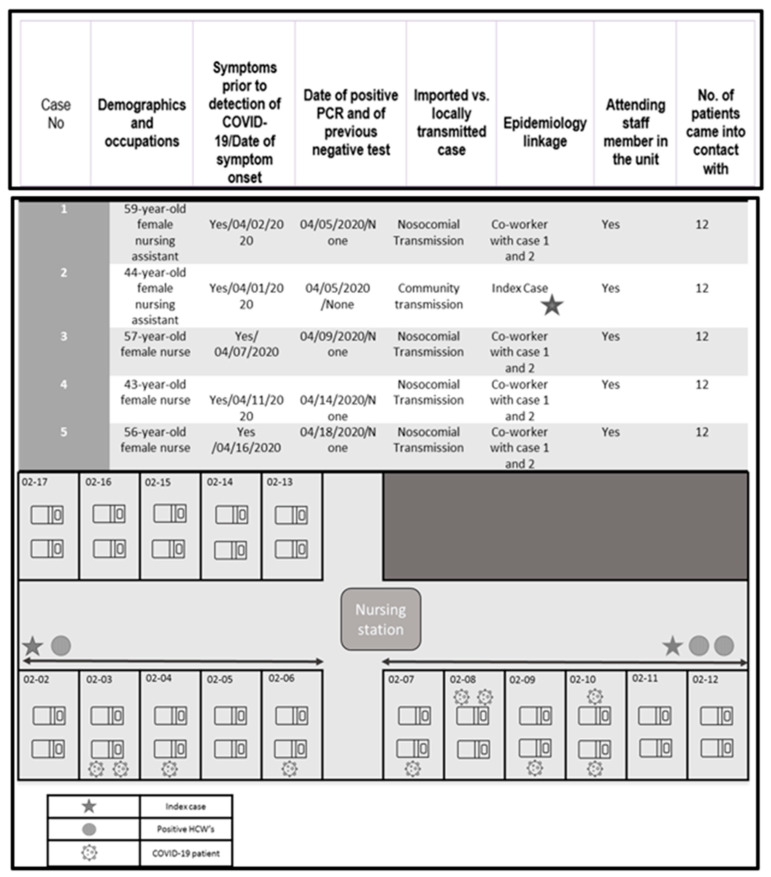
Distribution of HCWs and patients in the unit during the outbreak. Each nursing assistant and nurse had 12 patients under their care. The star shows the index case (nursing assistant). Additional information on the five HCWs with confirmed COVID-19 in the polyvalent ward is given in the box below the figure (demographic details, results of epidemiological investigation, and number of patients exposed).

The outbreak was declared on 7 April 2020. Based on the date of the positive PCR test, one of the infected HCWs was identified as the probable index case and was assumed to have acquired the infection in the community. After this date, a cluster of patients and HCWs from the same unit, all symptomatic, tested positive for COVID-19 by PCR. The distribution of all individuals is shown in Table 1 and Figure 2. The polyvalent ward consisted of 16 rooms and 32 beds (there were no private rooms).

The ward had a total staff of 43 HCWs (physicians, nurses, nursing and care staff, and cleaning staff) at the time of the outbreak. Of the 31 patients in the ward at this time, 10 patients, all in the nephrology section, tested positive for COVID-19. They had all been COVID-19-negative on admission. Five of the HCWs tested positive. The characteristics of the patients and HCWs are shown in Table 1.

### 3.3. Outbreak Management

Once the outbreak had been identified, the following control measures were taken:

- Quarantine (14 days of preventive isolation for close-contact HCWs);
- Transfer of positive patients to COVID isolation ward;
- Implementation of preventive droplet and contact measures for all patients remaining in the ward, applicable to anyone entering the room: hand hygiene and use of surgical mask (or FFP2 mask for aerosol-generating procedures), eye protection (for aerosol-generating procedures or procedures with a risk of splash), gown (and apron for splash protection), and gloves;
- Twice-weekly training sessions for all HCWs linked to the ward to emphasize the importance of preventing cross-infection and reinforce proper use of personal protection equipment (PPE) and hand hygiene. Before the COVID-19 pandemic, this ward had high compliance of infection control measures: great managing with isolation precautions, appropriate glove use, good hand hygiene compliance rate (2017: 87%; 2018: 87%; 2019: 69%), and when the pandemic started, universal masking was carried out for all HCWs; unfortunately, the infection control program was not available full time for conducting observations in non-COVID-19 units during the first part of the pandemic—in order to that, we have no data of compliance rates for this time;
- Enhanced cleaning and disinfection: regular cleaning, followed by disinfection with 0.1% sodium hypochlorite (1000 ppm) of patient rooms (once a day), frequently touched surfaces, and all equipment in the affected ward (twice a day);
- Weekly PCR tests for patients and HCWs on the ward;
- Testing of proper operation of ventilation systems and adequate supplies of acceptable-quality indoor air adapted to occupancy at any given time;
- These measures controlled the outbreak, and no new cases were detected up to the end of the study period (31 May).

## 4. Discussion

COVID-19 is primarily a community-spread disease with a risk of nosocomial transmission from visitors, HCWs, or patients who are incubating the infection, have a mild form of the disease, or are asymptomatic [14]. Hospital-based syndromic surveillance can facilitate the early detection of healthcare-associated outbreaks [23], and any surveillance system for HCWs must be supported by a comprehensive outbreak management strategy to achieve containment.

Response to outbreaks, however, is challenging due to the lack of research-based recommendations, particularly in the case of SARS-CoV-2, which is a novel pathogen that has spread rapidly throughout the world and caused community and hospital outbreaks [24].

Nosocomial infections have been documented in healthcare organizations that have strict contact and droplet precautions and use PPE designed to protect against droplet but not aerosol exposure.

We believe that the COVID-19 cases detected in the outbreak at our hospital might have been caused by airborne transmission of the virus from presymptomatic HCW carriers since family visits were forbidden during the study period. In other scenarios, hospital-acquired cases could result from asymptomatic infections or false-negative PCR results [25].

Of the 46 HCWs in the study ward, 5 (11%) tested positive for COVID-19, and they all had compatible clinical symptoms. Surveillance testing showed negative results for the remaining 41 HCWs. In other words, no asymptomatic carriers were identified. Prompt detection strategies such as routine PCR screening of HCWs, particularly in vulnerable areas, could help prevent disease spread.

Our outbreak illustrates how patients admitted to a COVID-19-free ward in a pandemic situation can become infected and possibly die. The mortality rate in our case was high, at 50%. This is similar to rates reported elsewhere [26] and may be linked to the patients’ advanced age and significant comorbidity (in our case, kidney disease). Nonetheless, in the observational COPE–Nosocomial Study, Carter et al. [27] concluded that nosocomial transmission was not associated with an increased risk of mortality and that it might even carry a lower risk than a community-acquired disease.

Our findings also provide further evidence that previous contact with healthcare facilities is common in patients who develop COVID-19 [19]. The actions taken by our hospital’s infection control team in response to the outbreak limited the number of hospital-acquired cases to 10 and prevented the spread of the virus to other non-COVID wards, where it could have had a similar impact. Interestingly, the outbreak was controlled in a short period (2 weeks) [28].

Hospitals need to create separate areas for patients with known or suspected COVID-19 and for those who are unlikely to have the disease [29]. In addition, strict monitoring for nosocomial acute respiratory illness is essential, especially in non-COVID-19 areas.

The IPC team has a crucial role in all hospitals since it is responsible for drawing up protocols on PPE use and hand hygiene monitoring, providing IPC training for all staff, and ensuring adequate implementation of measures taken. The importance of regularly cleaning and disinfecting all surfaces and medical equipment between patient contacts to reduce fomite transmission and of ensuring proper ventilation should be stressed to all staff. Finally, because asymptomatic infected patients can transmit the virus, consideration should be given to routine weekly PCR testing of all frontline staff, and possibly even patients in non-COVID wards, regardless of whether or not there is an outbreak [25]. Daily surveillance rounds to monitor compliance with preventive measures are also recommended.

The COVID-19 pandemic constitutes an unprecedented challenge for healthcare systems and communities worldwide. We hope that the lessons learned from this nosocomial outbreak at our hospital with a negative PCR for COVID-19 patients will help strengthen measures to protect against such outbreaks and significantly reduce the risk of in-hospital transmission. We have described how our hospital implemented infection control measures on a COVID-19-free ward at the peak of the pandemic, and how the entire hospital was forced to implement major changes in a short time. Hospitals should implement contingency plans that include IPC protocols for COVID-19-free areas.

## Figures and Tables

**Table 1:** Clinical and demographic characteristics of patients and healthcare workers with COVID-19 by disease severity and survival.

	SARS-CoV-2-Positive Hospitalized Patients (*n* = 10/31)	SARS-CoV-2-Positive Healthcare Workers (*n* = 5/43)
Age * years, mean (SD)	68.7 (15.5)	52 (7.8)
**Sex**		
Male	5 (50%)	-
Female	5 (50%)	5 (100%)
Days from admission to positive PCR, median (IQR)	10.5 (6–22)	
**Underlying diseases**		
No comorbidities	2 (20)	5 (100%)
Charlson Comorbidity Index ≥ 2	8 (80)	0
**Clinical presentation**		
**Symptoms**		
Fever	7 (70%)	5 (100%)
Cough	5 (50%)	3 (60%)
Fatigue	8 (80%)	3 (60%)
Dyspnea	7 (70%)	0
Diarrhea	2 (20%)	2 (40%)
Asymptomatic	2 (20%)	0
Radiographic findings	7 (70%)	0
**All-cause mortality**		
Died	5 (50%)	0
Alive	5 (50%)	5 (100%)
**Mortality due to COVID-19**		
No	1 (10%)	-
Yes	4 (40%)	-

* Statistically significant difference. Data are presented as *n* (%) unless otherwise specified. SARS-CoV-2 severe acute respiratory syndrome coronavirus 2, COVID-19 coronavirus disease-19, SD standard deviation, IQR interquartile range.

## Data Availability

The authors confirm that the data supporting the findings of this study are available within the article. Demographic, clinical, laboratory, and epidemiological data were collected from hospital and primary care electronic medical records.
